# Conserved sperm factors are no longer a bone of contention

**DOI:** 10.7554/eLife.68976

**Published:** 2021-04-28

**Authors:** Xue Mei, Andrew Singson

**Affiliations:** Waksman Institute of Microbiology and Department of Genetics, Rutgers UniversityPiscatawayUnited States

**Keywords:** fertilization, spermatozoon, oocyte, gamete fusion, gamete interaction, Mouse

## Abstract

Proteins related to a molecule involved in the formation of osteoclasts in bone are required for fertilization in worms, flies and mammals.

**Related research article** Inoue N, Hagihara Y, Wada I. 2021. Evolutionarily conserved sperm factors, DCST1 and DCST2, are required for gamete fusion. *eLife* **10**:e66313. doi: 10.7554/eLife.66313

Osteoclasts are multinucleated cells that break down bone for skeletal maintenance, repair, and remodeling. Experiments on mice have established that a gene called *DC-stamp* (dendritic cell-specific transmembrane protein) is involved in progenitor cells fusing to make osteoclasts (Kukita et al., 2004; Kodama and Kaito, 2020). Two related genes, *Dcst1* and *Dcst2* (DC-stamp domain containing 1 and 2), are expressed in the testes of mice, and likely share a common ancestor with a group of invertebrate genes required for fertilization (Mei and Singson, 2021), including *snky* in *Drosophila* (Wilson et al., 2006), and *spe-49* and *spe-42* in *C. elegans* (Kroft et al., 2005; Wilson et al., 2018). Together these sperm-specific genes span between 700 million and one billion years of evolutionarily conserved function.

Now, in eLife, Naokazu Inoue (Fukushima Medical University), Yoshihisa Hagihara (AIST) and Ikuo Wada (Fukushima Medical University) report that the DCST1 and DCST2 proteins are required for fertilization in mice (Inoue et al., 2021). After sperm have migrated to the egg, fertilization involves several stages: the spermatozoa must first interact with and penetrate the egg coat, and then adhere to the egg plasma membrane. Next, the plasma membrane of the sperm and egg must fuse to form a zygote. The sperm of male mice lacking the genes *Dcst1* and *Dcst2* can penetrate the egg coat, but they are unable to fuse: this indicates that these genes have a direct or indirect role in cell fusion that is reminiscent of the role of *DC-stamp* in osteoclast formation.

Comparing DCST1 and DCST2 to related invertebrate and human proteins, Inoue et al. found that mouse DCST1 was most closely related to human DCST1, nematode SPE-49 and fruit fly SNKY, whereas mouse DCST2 was closer to human DCST2, nematode SPE-42 and fruit fly DCST2. Single-gene knockouts of *Dcst1* and *Dcst2*, as well as double-knockout mice, exhibited male-specific sterility, with mutant spermatozoa failing to fertilize eggs in vitro. The spermatozoa from the double knockouts could reach the egg and undergo the acrosome reaction to penetrate the egg coat, but then they accumulated in the region between the egg coat and the egg membrane (Figure 1).

**Figure 1.**
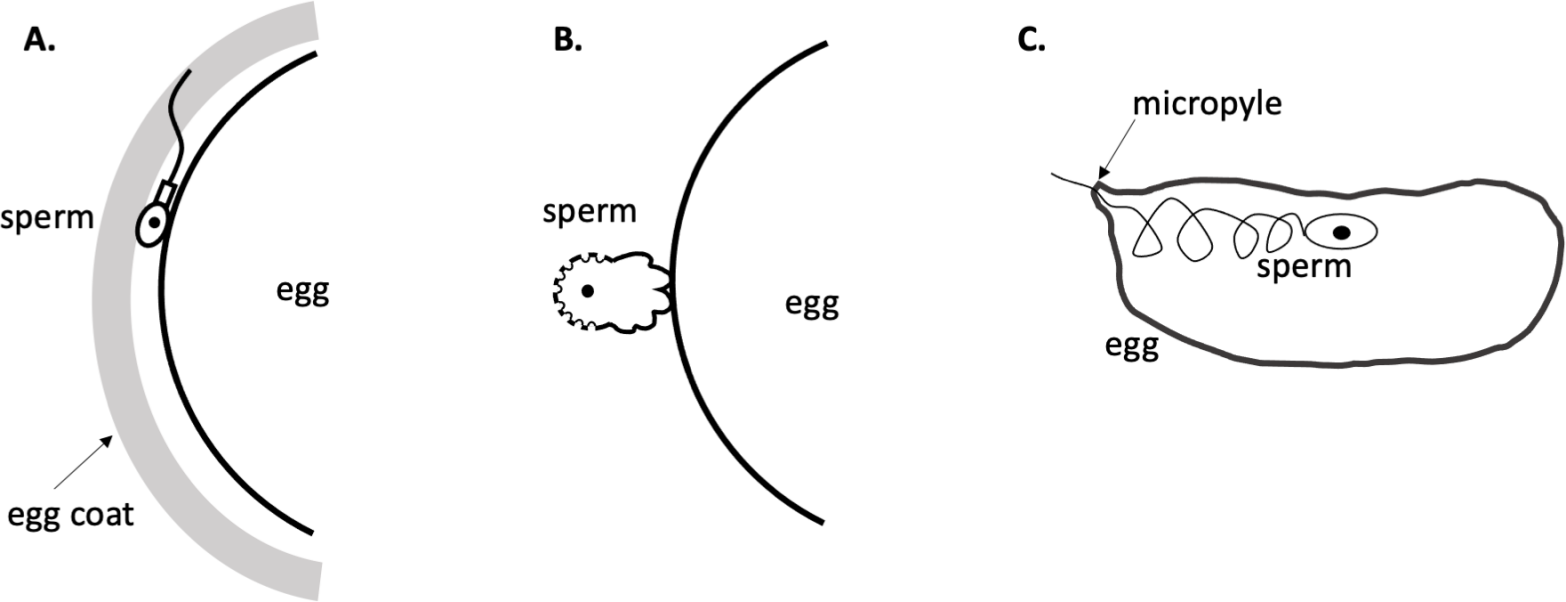
The interruption of fertilization in *Dcst*-related gene mutants in different species. (**A**) In mammals, sperm mutant for *Dcst1* and/or *Dcst2* can penetrate the egg coat and contact the egg plasma membrane, but they do not fuse with the egg. (**B**) In *C. elegans*, *spe-42* or *spe-49* mutant sperm can contact the egg plasma membrane, but they do not fuse with the egg. (**C**) In *Drosophila*, giant sperm enter the egg through a small opening called a micropyle: however, in *snky* mutant sperm the plasma membrane of the sperm does not break down, thus blocking nuclear fusion.

These results indicate that DCST1 and 2 are not required for sperm migration to egg, the acrosome reaction, or penetration of the egg coat. In fact, the phenotype of the mouse double mutant is similar to that of mice lacking other key sperm molecules during fertilization, including the immunoglobulin superfamily proteins IZUMO1 and SPACA6. These two proteins are involved in sperm-egg recognition, adhesion or fusion (Bianchi and Wright, 2020). When spermatozoa mutant for both *Dcst1* and *Dcst2* contacted the egg in vitro, it appeared that IZUMO1, its egg-surface binding partner JUNO, and an egg-surface molecule called CD9, were all recruited normally to the interface between the sperm and the egg. This suggests that key molecules are recruited normally despite fusion failing.

Inoue et al. next investigated the presence of IZUMO1 and SPACA6 in sperm mutant for different molecules. IZUMO1 was present in sperm lacking *Dcst1* and *Dcst2*, and also in sperm mutant for *Spaca6*. SPACA6, on the other hand, was lost in *Izumo1*, *Dcst1*, and *Dcst2* mutant sperm. These results suggest that these proteins, which are all needed for sperm-egg fusion, likely assemble in a hierarchical fashion, with IZUMO1 being assembled independently of other molecules (Krauchunas et al., 2016). Further analyses of these proteins in various mutant backgrounds may provide new insights into how they assemble and interact during fertilization.

The groundbreaking work of Inoue et al. suggests important future questions. Why are two similar proteins both required non-redundantly for fertility in worms and mammals? Precise protein localization, domain swapping, and studies examining the relationship between structure and function could shed light on this question. Additionally, the biochemical role of these proteins is not clear. It is possible that they act as signaling molecules with an unknown ligand (Chiu et al., 2017). However, loss of function phenotypes appear consistent with some role in either membrane fusion (in mammals and worms) or in membrane breakdown (in flies; Figure 1).

DCST1 and DCST2 and related proteins could be better understood by investigating the molecules they interact with. For instance, it has been shown in *C. elegans* that SPE-42 binds to other sperm membrane proteins involved in spermatogenesis and fertilization (Marcello et al., 2018). Its interaction with the dysferlin FER-1 is particularly intriguing, since FER-1 regulates calcium-mediated membrane fusion during worm spermatogenesis (Washington and Ward, 2006). Mutations in a human dysferlin gene are associated with limb-girdle muscular dystrophy due to a loss of membrane repair in skeletal muscles (Bashir et al., 1998). This indicates that, in addition to a better understanding of fertilization, ongoing work on genes related to *Dcst1* and *Dcst2* may provide new insights into muscle and bone health.

In most species, relatively few gamete interaction molecules have been genetically defined (Mei and Singson, 2021), so the existence of conserved gamete interaction genes between invertebrates and mammals has been ‘a bone of contention’. A manuscript recently posted on bioRxiv confirms the role of *Dcst1* and *Dcst2* in male fertility described by Inoue and co-workers (Noda et al., 2021). This paper further demonstrates that zebrafish *dcst1/2* are also required for fertilization. The characterization of genes related to sperm *Dcst1* and *Dcst2* in diverse species, including humans, should go a long way towards ending debates over deeply conserved gamete function genes. As the pace of fertility gene discovery increases in both vertebrate and invertebrate model systems, we fully expect that more fundamental molecular parallels and key features of the interaction between sperm and egg will be discovered.
